# Man Presenting After Hydrochloric Acid Ingestion

**DOI:** 10.5811/cpcem.1436

**Published:** 2023-12-06

**Authors:** Emma R. Furlano, Gregory P. Wu, Brendan Vosburgh, Cameron R. Waldman, Jessica Noonan, Alexander Bracey

**Affiliations:** * Albany Medical Center, Department of Emergency Medicine, Albany, New York; † Albany Medical Center, Department of Medicine, Division of Pulmonary Critical Care Medicine, Albany, New York; ‡ Albany Medical Center, Department of Emergency Medicine, Division of Toxicology and Addiction Medicine, Albany, New York

**Keywords:** *caustic ingestion*, *toxicology*, *acidic ingestion*, *critical care*, *esophageal perforation*

## Abstract

**Case Presentation:**

We describe a case of a man who developed severe caustic injury of his upper gastrointestinal tract after ingestion of a commercially available 9.5% hydrochloric acidic cleaning solution. He rapidly deteriorated and required endotracheal intubation. He underwent several imaging modalities demonstrating his injuries and ultimately succumbed to his injuries.

**Discussion:**

Acidic caustic ingestions may range in severity and uncommonly result in death. Diagnosis is most often achieved by esophagogastroduodenoscopy, although computed tomography may increasingly play a role in defining the extent of injury. Esophagogastroduodenoscopy findings are often assigned a Zargar grade, which guides management. Medical management of acidic caustic ingestion may include bowel rest, steroids, antibiotics, and proton pump inhibitors depending on the extent of injury, although surgery may be required if esophageal perforation occurs.

CPC-EM Capsule What do we already know about this clinical entity?

*Acidic ingestions can cause injury from coagulative necrosis, especially of the GI tract. Severe ingestions can cause viscous perforation and multisystem organ failure.*

What is the major impact of the image(s)?

*EGD is the gold standard for injury diagnosis and management planning, but the role of CT is expanding for acidic ingestions. EGD is contraindicated in viscous perforation.*

How might this improve emergency medicine practice?

*When available, bedside EGD can be used for rapidly determining patient management plans. CT is a useful adjunct for surgical planning and in viscous perforation.*

## CASE PRESENTATION

**Image 1.**
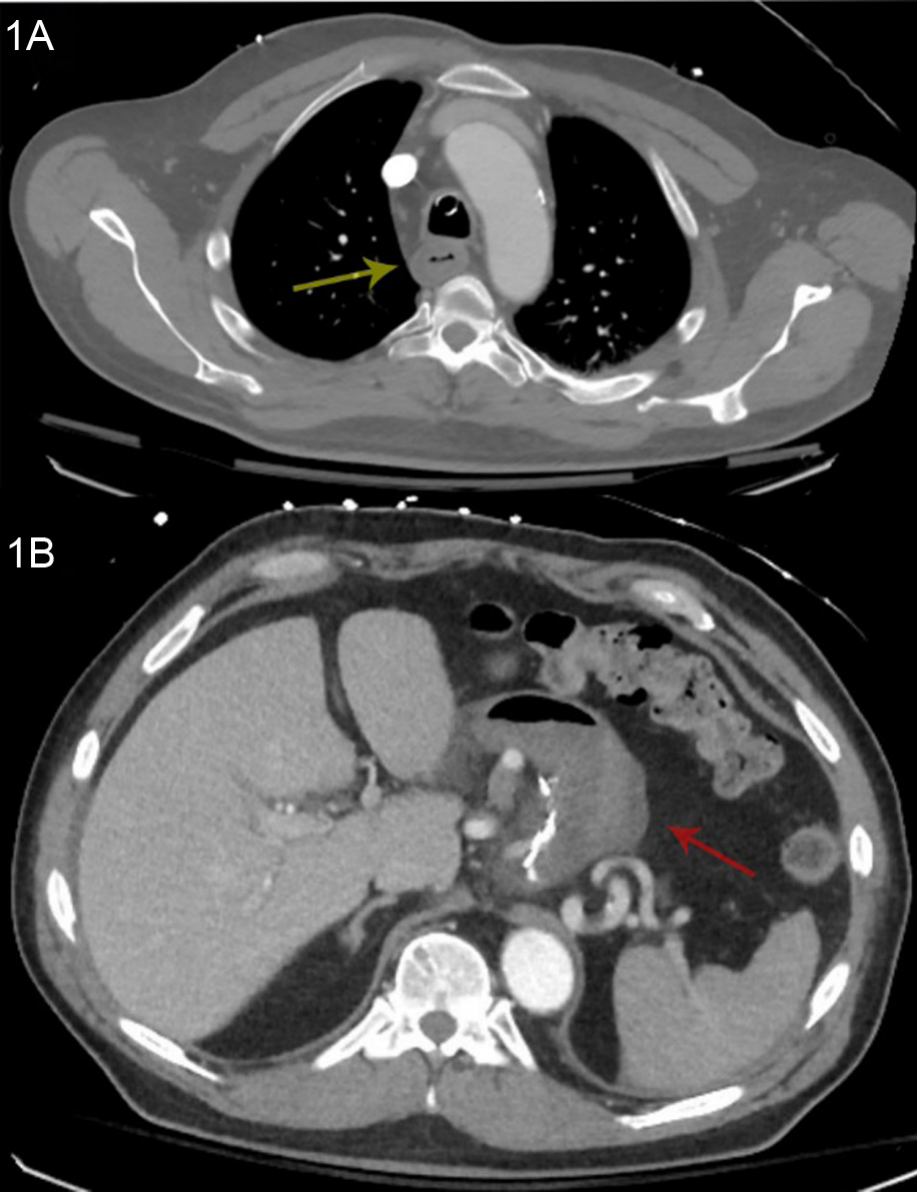
Computed tomography (CT) of the chest and abdomen shortly after admission. 1A: CT chest with contrast shortly after intubation; the yellow arrow indicates region of esophageal thickening in the proximal esophagus. 1B: CT abdomen pelvis with contrast shortly after intubation; the red arrow shows thickening of the stomach.

**Image 2.**
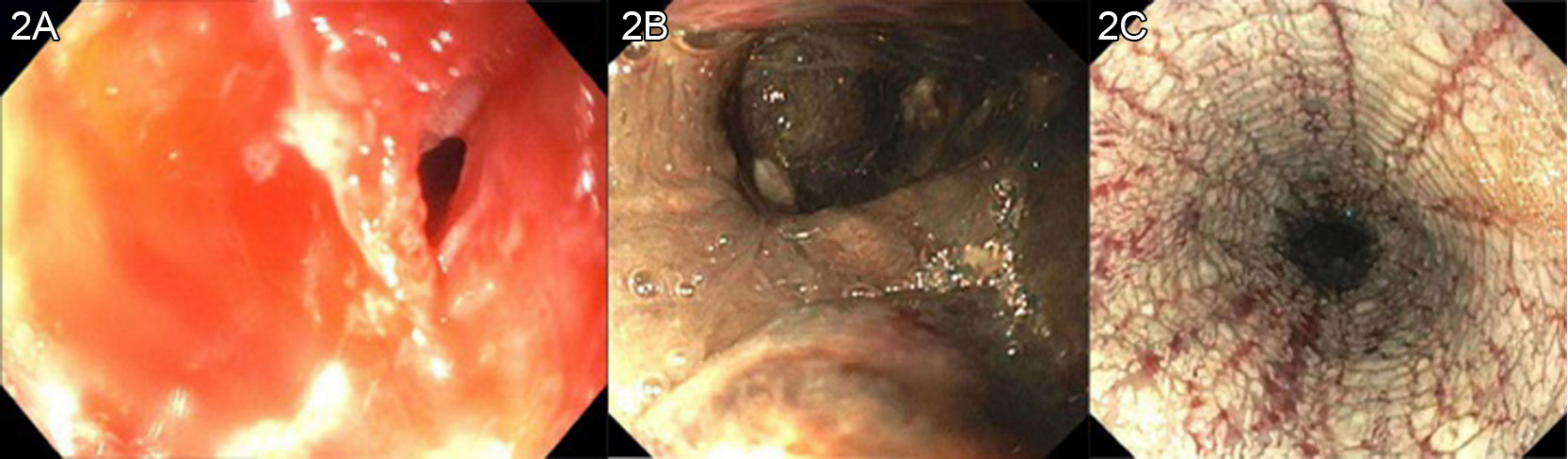
Endoscopy in the first 24 hours after intubation. 2A: view of the proximal esophagus showing diffuse thickening and edema, with narrowed lumen from caustic esophagitis. 2B: view of the lower third of esophagus showing grade 3B esophagitis. 2C: view of the duodenum, with hematin and caustic injury.

A 63-year-old man presented to the emergency department (ED) via ambulance with recurrent hematemesis after an intentional ingestion of a commercially available 9.5% hydrochloric acid, toilet bowl cleaning solution. While initially stable, he rapidly deteriorated following arrival and was unable to tolerate his secretions and ultimately required endotracheal intubation for airway protection. He underwent computed tomography (CT) of his chest, abdomen, and pelvis (Image 1), which identified diffuse thickening of the proximal gastrointestinal (GI) tract without evidence of perforation. A pantoprazole infusion was initiated while gastroenterology and toxicology were consulted. A bedside esophagogastroduodenoscopy (EGD) was performed in the ED, which revealed Zargar grade 3B esophagitis and active bleeding from the duodenum (Image 2).

**Image 3.**
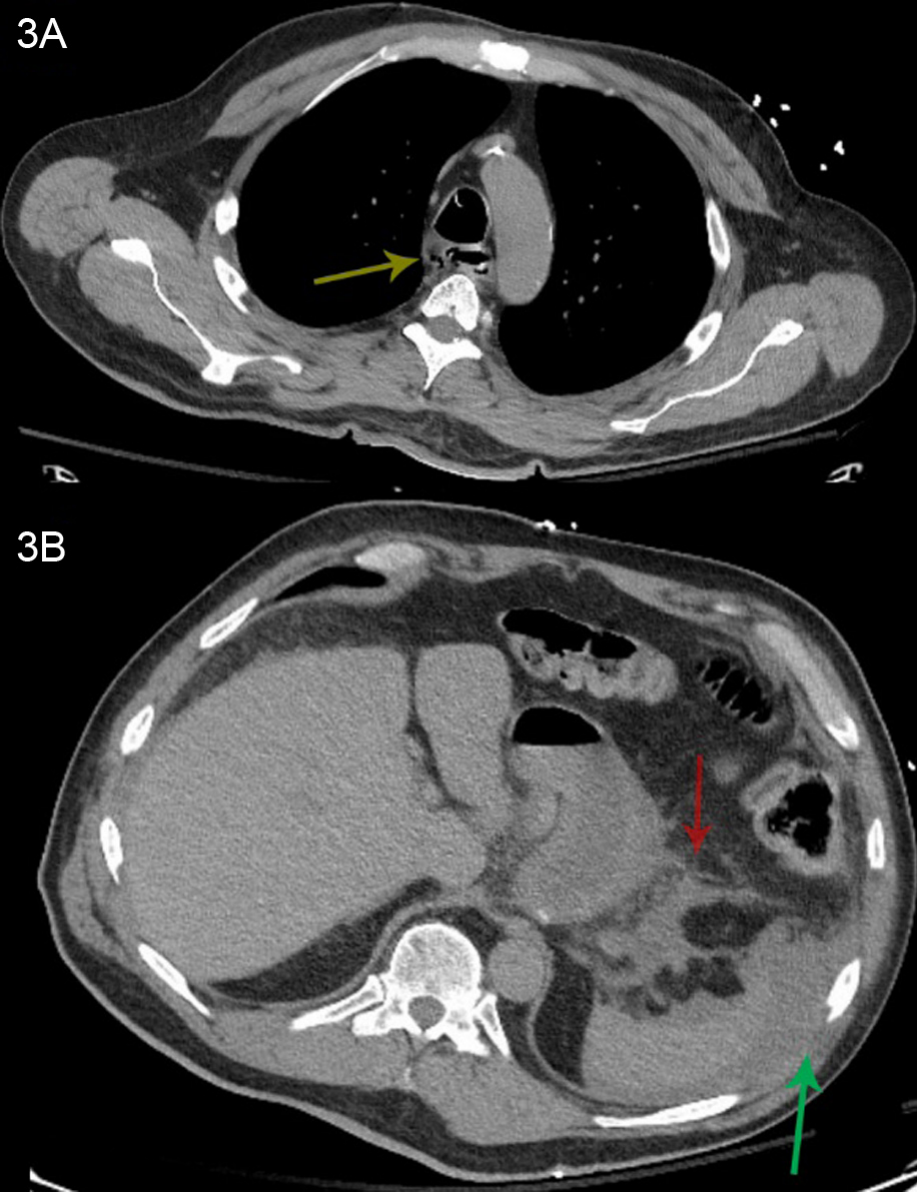
Computed tomography (CT) of the chest and abdomen one day later. 3A: CT chest without contrast, same region cut as 1A. The yellow arrow indicates mediastinal air around the esophagus. 3B: CT abdomen pelvis without contrast, same region as 1B. The red arrow indicates free fluid around the stomach; the green arrow indicates free fluid around the spleen.

The patient was admitted to the intensive care unit for ongoing medical management. Repeat CT imaging revealed esophageal perforation with mediastinal and intra-abdominal free fluid (Image 3). Given the extent of the esophageal injury, the patient was not a candidate for esophagectomy and ultimately died despite aggressive medical management.

## DISCUSSION

Hydrochloric acid ingestion resulting in death is rare. In 2019, the American Association of Poison Control Centers reported 184,677 exposures to cleaning products, which includes caustics. Of 16 deaths related to acidic ingestion, only two were from hydrochloric acid, and neither one was related to toilet cleaning products.^1^ Acidic ingestions cause a coagulative necrosis in the GI tract. Severe oropharyngeal pain, odynophagia, and hypersalivation are commonly observed clinical features following ingestion, although, importantly, the degree of injury does not correlate with symptom severity.^2^ While EGD remains the gold standard, the role of CT for diagnosis, prognostication, and surgical planning is expanding. In addition to diagnosis, EGD can be used to assign a Zargar grade of esophageal injury.

The Zargar grading system is widely used for caustic ingestion grading and guides further management.^3^ ^–^ ^5^ Zargar grade 2A or less may be treated conservatively with bowel rest and supportive care. High-dose systemic corticosteroids are indicated for the prevention of strictures in Grade 2B lesions, while more severe injury may require surgical intervention. However, EGD has limited ability to detect the depth of necrotic injury, which may influence surgical planning.^5^ Esophagogastroduodenoscopy is ideally performed within the first 48 hours from time of injury to minimize the risk of iatrogenic esophageal perforation and is contraindicated in instances of known viscous perforation.^4^ ^,^ ^6^ Multiple grading systems to compare CT findings to EGD findings have been proposed; in one, CT grade I lesions correspond to Zagar grade 1–2A, while CT grade III corresponds to Zargar grade 3B.^5^ ^–^ ^7^

Blind insertion of naso- or orogastric tubes is contraindicated, as this may result in further esophageal injury or perforation, but they can be placed under direct endoscopic guidance.^4^ Antibiotics for identified sources of infection and proton pump infusions are reasonable with consequential injuries.^8^
